# Estimating the budget impact of orphan drugs in Sweden and France 2013–2020

**DOI:** 10.1186/1750-1172-9-22

**Published:** 2014-02-13

**Authors:** Adam Hutchings, Carina Schey, Richard Dutton, Felix Achana, Karolina Antonov

**Affiliations:** 1 Dolon Ltd, 175-185 Gray’s Inn Road, London WC1X 8UE, UK; 2 GMAS Sàrl, Route de Buchillon 65, St-Prex 1162, Switzerland; 3 KeyFormula Ltd, Roma House, Charrington Place, St Albans, Hertfordshire AL1 3NX, UK; 4 Biostatistics Unit, University of Leicester, University Road, Leicester LE1 7RH, UK; 5 LIF, Box 17608 Stockholm 118 92, Sweden

**Keywords:** Orphan medicines, Budget impact, Health economics, Europe

## Abstract

**Background:**

The growth in expenditure on orphan medicinal products (OMP) across Europe has been identified as a concern. Estimates of future expenditure in Europe have suggested that OMPs could account for a significant proportion of total pharmaceutical expenditure in some countries, but few of these forecasts have been well validated. This analysis aims to establish a robust forecast of the future budget impact of OMPs on the healthcare systems in Sweden and France.

**Methods:**

A dynamic forecasting model was created to estimate the budget impact of OMPs in Sweden and France between 2013 and 2020. The model used historical data on OMP designation and approval rates to predict the number of new OMPs coming to the market. Average OMP sales were estimated for each year post-launch by regression analysis of historical sales data. Total forecast sales were compared with expected sales of all pharmaceuticals in each country to quantify the relative budget impact.

**Results:**

The model predicts that by 2020, 152 OMPs will have marketing authorization in Europe. The base case OMP budget impacts are forecast to grow from 2.7% in Sweden and 3.2% in France of total drug expenditure in 2013 to 4.1% in Sweden and 4.9% in France by 2020. The principal driver of expenditure growth is the number of new OMPs obtaining OMP designation. This is tempered by the slowing success rate for new approvals and the loss of intellectual property protection on existing orphan medicines. Given the forward-looking nature of the analysis, uncertainty exists around model parameters and sensitivity analysis found peak year budget impact varying between 2% and 11%.

**Conclusion:**

The budget impact of OMPs in Sweden and France is likely to remain sustainable over time and a relatively small proportion of total pharmaceutical expenditure. This forecast could be affected by changes in the success rate for OMP approvals, average cost of OMPs, and the type of companies developing OMPs.

## Background

Rare diseases are life-threatening or severely debilitating conditions that affect very small patient populations. It is estimated that 6,000 to 8,000 rare diseases affect approximately 30 million people in the European Union (EU) [1]. Rare diseases, as defined according to the European Orphan Medicinal Product (OMP) Regulation, affect no more than five in 10,000 individuals [2].

The OMP Regulation was adopted in 1999 and came into force in 2000 with the aim of establishing regulatory pathways and incentives for companies to develop treatments for life-threatening or chronically debilitating conditions. Overall, the OMP Regulation has been perceived to be a great success [3]. Prior to the Regulation, very few treatments were available for rare diseases, whereas in December 2012, 878 drugs had received an OMP designation, including 78 drugs with a marketing authorization in the EU [4].

After the introduction of the OMP Regulation in Europe, and since the approval of the first OMPs, health policy makers and payers have raised concerns about their cost, both at a patient level and in aggregate across diseases [5,6]. Concerns about the pricing, affordability and cost-effectiveness of OMPs have led to calls to revisit the incentives laid out in the OMP Regulation, and to adopt more stringent pricing and reimbursement policies for OMPs [7]. For instance, the Dutch Health Care Insurance Board (CVZ) has questioned whether it is reasonable to reimburse drugs for rare lysosomal storage disorders due to concerns about cost effectiveness [8].

Several studies conducted in European countries have estimated the market availability or budget impact of OMPs in different settings [9,10]. A study observed that there was substantial variation in the market uptake of OMPs across the 23 countries included in the analysis. The gross domestic product and the availability of a formal health technology assessment organization within each country proved to be key determinants in the uptake of OMPs [11]. Because the decision to fund specific OMPs is within the remit of each country, access to approved OMPs varies greatly across Europe [12]. An analysis of OMP budget impact in five European countries in 2007 found the total pharmaceutical expenditure on existing OMPs is very low in Europe (1.0–2.1%, depending on the country) [10]. A previous study by the authors estimated the average annual cost of OMPs per patient to predict the total cost of orphan medicines in Europe between 2010 and 2020 as a percentage of total European pharmaceutical expenditure, and concluded that concerns of spiraling OMP expenditure were not justified [13].

Although these studies inform on interesting aspects of OMPs, they are general in their approach and mainly report retrospective data. In order to address concerns that the development of new OMPs could affect future healthcare budgets, analyses incorporating rigorous methodology are warranted. To date, the OMP markets of Sweden and France have not been thoroughly analyzed as stand-alone markets. OMP expenditure in these two countries was investigated in this study because Sweden and France respectively are considered to be at the restrictive and permissive ends of the spectrum with regards to OMP accessibility and reimbursement [14-16].

This study developed a dynamic forecasting model to examine historical trends in OMP designation, market authorization, sales, and budget impact in these two markets, from 2000 to 2012. These historical data were then used to predict the evolution in OMP use for existing diseases and new indications between 2013 and 2020, with a view to estimating their budget impact on the national pharmaceutical market. The analysis establishes a robust forecast of the future budget impact of OMPs on the French and Swedish healthcare systems.

## Methods

**Figure 1:**
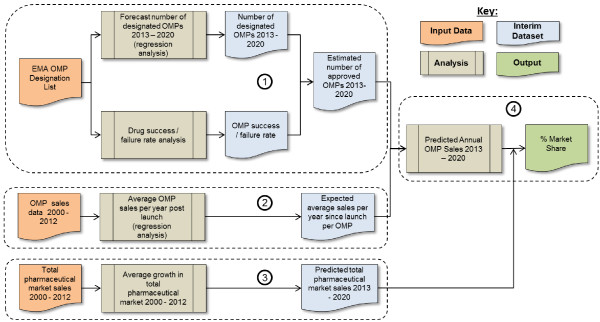
OMP budget impact forecast model structure (2013–2020).

For the period 2000–2012, the OMP budget impact was calculated for each country based on the observed sales of authorized OMPs as a percentage of total pharmaceutical industry sales [17,18]. To estimate the corresponding budget impact for the 2013–2020 period, a dynamic forecasting model encompassing four sub-analyses was created (Figure 1).

### Sub-analysis 1. Estimated number of approved OMPs in the period 2013–2020

The number of new OMP marketing authorization approvals was predicted by applying a time-dependent ‘success rate’ factor to the pool of designated OMPs that are currently unauthorized, which may potentially be authorized as OMPs in the future.

New OMP designations for the period 2013 – 2020 were estimated based upon a linear regression of historical designation rates per year since the introduction of the OMP Regulation. The predicted new OMP designations were added to those OMPs already designated in a time-dependent matrix that specified the ‘age’ (years since designation) of each drug at each year in the model. The number of designated OMPs with approved market authorization was estimated using an age-dependent success rate factor calculated for each year following OMP designation, based on an analysis of all OMPs designated and approved in Europe since 2000 [4]. Each designated OMP’s lifecycle was analyzed, noting its status in each year since designation (Pending Approval, Approved, Withdrawn). An age-specific success rate was calculated across the whole sample for each year following OMP designation. With this methodology, the issue of lag times between designation and approval was accounted for in the analysis. As well as new drugs achieving OMP designation, drugs with an existing designation can have that status withdrawn, possibly because the drug failed during clinical development, was developed for a non-orphan indication or for commercial reasons. To account for this, a similar distribution was estimated to predict the likelihood of a designated OMP being withdrawn from the designation list at each year since designation.

The analysis was then conducted on different time periods to determine if the success rate was constant over time. In order to select the appropriate distribution to predict the success rate from 2013 onwards, an identical analysis was undertaken on orphan drug data from the United States (US) [19], to corroborate any temporal trends seen in Europe, since orphan drug legislation was introduced in the US as early as 1983 [20].

### Sub-analysis 2. Expected average sales per year since launch per OMP

To calculate the average sales per year since launch for an OMP in Sweden and France, sales data of all OMP authorized in Europe since 2000 were analyzed separately for each market. Sales data for these OMPs in each country were obtained for 2000–2012 [17,18] and quantified by year post-launch. A regression analysis estimated the average sales for each year post-launch across all OMPs. The regression model was fitted in WinBUGS [21]. The model specified the relationship between mean sales and year post-launch taking into account correlations between year-on-year sales for the same OMP (within drug variance), between sales of different OMPs (between drug variance), and between mean sales and time effects (random slopes and intercepts). Several models were fitted, with best fit being determined using the Deviance Information Criterion (DIC). The model with the lowest DIC was judged to be the best fit. Based on the DIC, the best fitting model was a random intercept and slope model with log (year) and year-squared as time-dependent variables.

The effect of loss of intellectual property protection (IPP) for OMPs was built into the forecast by adjusting each OMP average annual sales. A patent lawyer analyzed patents and marketing exclusivity rights of OMPs marketed in Europe [22]. The most extensive form of IPP (patent, supplementary patent, marketing exclusivity) was used for each OMP. The average duration of IPP was 12 years. The average sales were adjusted within the model to reflect the expected price change at loss of IPP. Price cuts of 65% and 60% were applied on loss of IPP in Sweden and France, respectively, based on market-specific price dynamics [23,24].

### Sub-analysis 3. Predicted total individual pharmaceutical market value (for Sweden and France)

In order to provide a relative estimate of the OMP budget impact, it was necessary to predict the total pharmaceutical market growth rate for each country until 2020. This was estimated using Intercontinental Medical Statistics (IMS), Groupement pour l’Elaboration et la Réalisation de Statistiques (GERS) sales data for France, and Apotekets Inköps *Pris* (AIP) pharmacy-in prices for Sweden to ensure alignment with the OMP market estimate from 2000–2012 [17,18,25].

### Sub-analysis 4. Predicting the budget impact of OMPs 2013–2020

Results from Sub-analysis 1 (number of OMPs receiving marketing authorization each year, and age of OMP at that time point) and Sub-analysis 2 (average expected sales for an OMP at given age) were combined to estimate the total sales of OMPs from 2013 through to 2020. The relative budget impact was reached by dividing this gross estimate by the predicted total pharmaceutical market value in both markets over the same period (Sub-analysis 3).

Sensitivity analyses were undertaken on key factors in the model to test parameter uncertainty: change in price at loss of IPP, predicted market growth rate, success rate for designated OMPs obtaining marketing authorization approval, predicted growth in new OMP designations, and average total OMP sales post-launch.

## Results

### Estimated number of approved OMPs in the period 2013–2020

**Figure 2:**
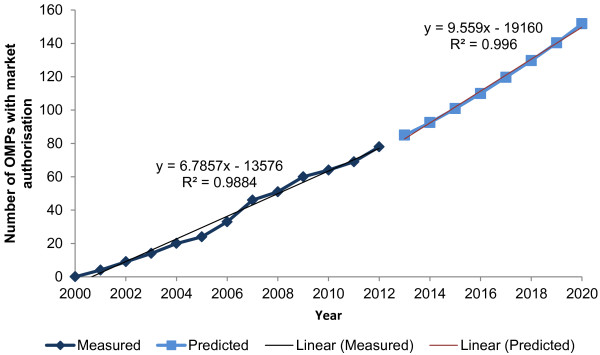
OMPs with marketing authorization in Europe: observed and predicted data.

**Figure 3:**
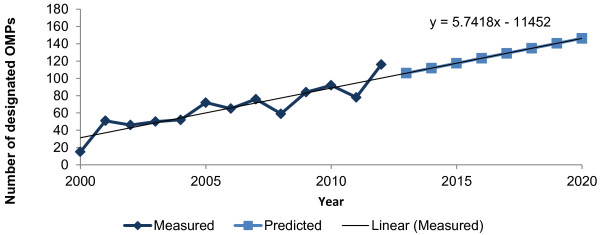
Observed and predicted new OMP designations per year in Europe.

The model predicts that by 2020, 152 OMPs will have marketing authorization in Europe (Figure 2). The predicted annual growth rate is higher than the observed trend over the last five years, reflecting the growing OMP designation rate (Figure 3).

While over 1,000 drugs have received OMP designation in Europe since 2000 [26], not all of these drugs have retained this status. The OMP designation of some drugs has been withdrawn, often because the drug development program has failed. In December 2012, 878 drugs currently had OMP designation in Europe, of which only 78 (8.8%) had obtained market authorization [4]. Manufacturers continuously apply to add New Chemical Entities to the list of designated OMPs, and the number of OMP designations has grown consistently since the introduction of the OMP Regulation in 2000, from 15 new designations in Europe per year in 2000 to 116 in 2012. A linear regression model (R^2^ = 0.79) fitted to the observed (measured) data predicted that there would be 146 new OMP designations per year by 2020 (Figure 3).

**Figure 4:**
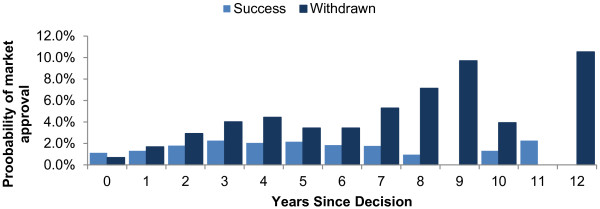
Annual market approval rates for designated OMPs in Europe.

**Figure 5:**
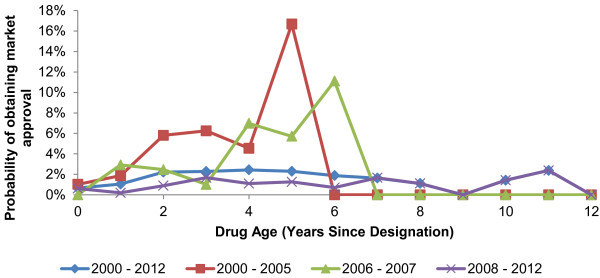
Success probability distributions for time periods since the introduction of European OMP Regulation in 2000 in Europe.

The annual success and failure probabilities by OMP age show that the chances of successful market authorization are low and constant over time, with the majority of successful drugs being approved between 2 and 7 years post designation (Figure 4). Since the introduction of the OMP Regulation, a trend towards lower market approval success rates over time was observed (Figure 5). The highest market approval success probabilities were recorded between 2000–2005 and the lowest between 2008 – 2012. (The lower success rates in more recent years do not reflect a lack of maturity in this data, as the success rates were calculated by dividing the number of approved drugs by the number of designated drugs *of the same age* during that period, not the number of designated products in the year of approval). Across all four time periods analyzed, the success rates all dropped around seven years following OMP designation. The analysis conducted on orphan drug data from the US [19] revealed a similar trend, whereby success rates fell in each consecutive decade from 1983 to 2012. Because of the age-specific nature of this analysis – accounting for time lags between designation and approval – this approach provides a more accurate estimate of success rates than simple ratios of approvals to designations within an individual year. Accordingly, the success distribution rate for the most recent European data (2008–2012) was felt to be the most likely approximation of future success rates in Europe, and the one that was used in the model to estimate the number of designated OMPs that would achieve marketing approval in future years.

### Expected average sales per year since launch per OMP

**Figure 6:**
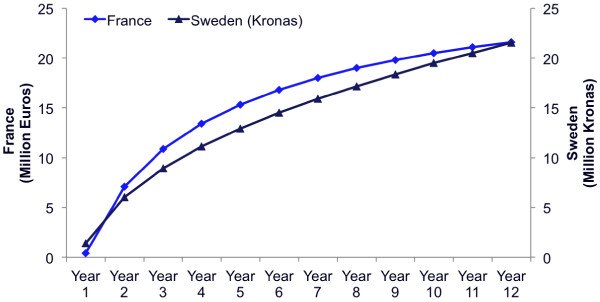
Modeled average OMP sales by year post-launch in France and Sweden.

According to the regression analysis, the modeled average OMP sales per year since 2000 grew at similar rates in both countries analyzed, up to seven years post-launch, after which sales progressed more slowly in France compared with Sweden (Figure 6). In the first four years post-launch, average sales grew to SEK 11.1 million in Sweden and EUR 13.4 million in France, and then the growth rate steadied, respectively reaching SEK 21.5 million in Sweden and EUR 21.6 million in France in year 12 post-launch. This reflects the relatively low sales that OMPs usually obtain, with the exception of a few high-cost OMPs such as imatinib. In France 64% of OMPs have sales of less than EUR 10 million [17,18].

### Predicted total individual pharmaceutical market value

**Figure 7:**
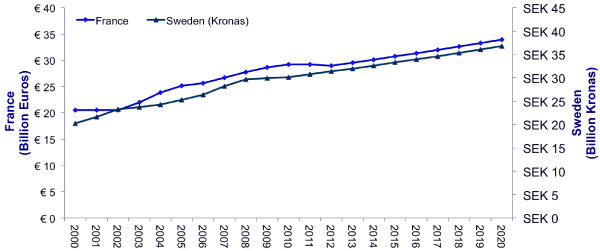
**Pharmaceutical market growth.** French and Swedish data are each observed for 2000–2012 [17,18]. Both countries are modeled forward from 2013 using a 2% per annum growth assumption.

Overall, during the 2000–2012 period, the Swedish pharmaceutical market grew from SEK 20.3 billion to SEK 36.8 billion [17], an average annual growth rate of 4.0%. During this period, the average annual growth rate for France was 3.0% (from EUR 20.5 billion to EUR 34.0 billion) [18]. Relatively quick growth was observed up to 2007 (4.0–5.0%), after which pharmaceutical market growth rates slowed following the 2008 economic crisis. The Swedish market grew modestly between 2008 and 2010, and fell slightly in 2012. Similarly, the French market slowed down between 2010 and 2012. Both countries were modeled forward from 2013 using a 2.0% per annum growth assumption (Figure 7), which the authors felt was a conservative estimate, representing a mid-point between the long-term growth rate over the last 12 years and the market stagnation seen after the 2008 economic crisis.

### Predicted budget impact of OMPs 2013–2020

**Figure 8:**
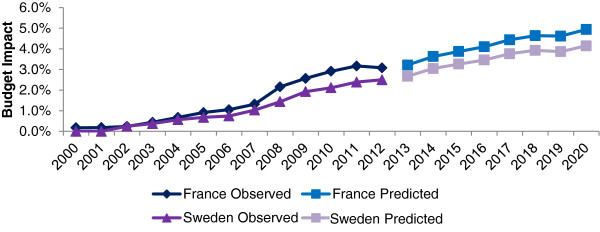
OMP budget impact as percentage of total pharmaceutical market: observed and predicted data.

The observed OMP budget impact in Sweden grew at a steady rate following the introduction of the OMP Regulation, reaching 0.7% of total drug sales in 2006. The budget impact growth rate accelerated between 2006 and 2009, then slowed more recently, reaching 2.5% of total pharmaceutical market value in 2012. The observed budget impact growth rate in France followed a similar trajectory to Sweden, although showing a more pronounced slowing in budget impact growth since 2008, with budget impact appearing to fall slightly in 2012 to 3.1% of total pharmaceutical sales (Figure 8). The model predicted that between 2013–2020 OMP budget impact will grow at a steady rate from 2.7% in Sweden and 3.2% in France in 2013 to 3.9% and 4.6% respectively by 2018, after which OMP sales will plateau at 4.1% in Sweden and 4.9% in France of the total pharmaceutical market (Figure 8).

### Sensitivity analysis

Because the predicted data are not simple extrapolations of observed data, but rather a modeled prediction incorporating additional information, sensitivity analyses were carried out (Table 1).

**Table 1:** Results from one-way sensitivity analyses on key model parameters

**Parameter**		Value*		**Peak year budget impact as% of total pharmaceutical spend (year)**	**Peak year budget impact as% of total pharmaceutical spend (year)**
				**Sweden**	**France**
1	Change in price at loss of IPP	Base	-65% (SE)	4.1% (2020)	4.9% (2020)
			-60% (FR)		
		Best	-80%	3.8% (2020)	4.4% (2020)
		Worst	0%	5.7% (2020)	6.4% (2020)
2	Pharmaceutical annual market growth rate 2012–2020	Base	2%	4.1% (2020)	4.9% (2020)
		Best	4%	3.4% (2018)	4.2% (2020)
		Worst	0%	4.9% (2020)	5.8% (2020)
3	Success rate for designated OMPs obtaining market authorization approval	Base	Observed trend rate 2008–2012	4.1% (2020)	4.9% (2020)
		Best	50% of base case rate	3.3% (2017)	3.9% (2017)
		Worst	Observed trend rate 2000–2005	9.0%	11.0% (2020)
4	Annual growth in new OMP designations from 2012	Base	y = 5.7418x - 11452	4.1% (2020)	4.9% (2020)
		Best	50% < base	4.0% (2020)	4.8% (2020)
		Worst	50% > base	4.2% (2020)	5.0% (2020)
5	Average total annual sales of an OMP post-launch (year 1 – year12)	Base	As per Figure 6	4.1% (2020)	4.9% (2020)
		Best	-50% p. a.	2.0% (2020)	2.5% (2020)
		Worst	+50% p. a.	8.1% (2020)	9.9% (2020)

*‘Best’ and ‘worst’ cases are from the perspective of funders of OMPs, with ‘best’ representing the lower budget impact and ‘worst’ the higher.

There is considerable uncertainty around the likely impact of loss of IPP on prices of OMP in future. Accordingly the budget impact was assessed assuming that there were to be no change in OMP price at the point of loss of IPP. In this scenario peak year budget impact reaches 5.7% and 6.4% of total pharmaceutical sales in Sweden and France, respectively. The trajectory of the modeled data under this scenario almost superimposes that of the forecasted cumulative market authorizations (Figure 2).

It was also felt to be important to understand the impact of a change in the average cost per year of OMPs. If the average cost per year increased by 50% in future, the peak year budget impact reaches 8.1% and 9.9% of total pharmaceutical sales in Sweden and France, respectively.

Sensitivity analyses further explored the estimated growth rate of the two pharmaceutical markets through to 2020, the success rates for designated OMPs achieving marketing authorization approval, and growth in new designations per year from 2013. Of these only the success rate change had a material impact on the prediction. When the predicted success rate is that observed in the first five years following OMP Regulation the peak year budget impact is 9% in Sweden and 11% in France.

## Discussion

Because approximately 30 million individuals are affected by rare diseases in the EU [1], the Orphan Medicinal Products Regulation implemented in 2000 recognized the unmet need for rare disease treatments, and implemented incentives for companies to develop treatments for these diseases. The price of individual OMPs and the growth of aggregate OMP expenditure across Europe has since been raised as a cause for concern by payers and health policy makers. However, concerns about budget impact may be misguided, and based on forecasts that are predicated on assumptions that high rates of OMP designation will lead to equally high levels of OMP approvals.

Results from this robust analysis estimate that the future budget impact of OMPs in France and Sweden will be sustainable, with peak budget impacts amounting to 4–5% of the total pharmaceutical expenditure by 2020. The budget impact is predicted to slow from 2018 onwards, as OMP growth rates converge with those of the overall pharmaceutical market.

There are two reasons why budget impact is expected to slow despite continued growth of OMP designations: first, the low market approval success rate for OMPs (Figure 4), and second, the savings from existing products losing IPP and being replaced by generic versions.

The success rates for OMP authorizations in Europe are unlikely to return to those levels reached in the early years post-Regulation, as per the worst-case scenario explored in sensitivity analysis. Early success rates in Europe were inflated by manufacturers of OMPs already available in the US taking advantage of newly introduced EU legislation to obtain approvals in Europe. European OMP authorization rates have since fallen (Figures 4, 5), reflecting trends in US orphan drug approval since 1983 [19]. It is more likely that future success rates will be lower than those used in our model. The scenario of 50% increase in average OMPs sales per year would represent a significant departure from the observed evidence to date.

A previous study undertaken by EURORDIS in 2009 also attempted to forecast the number of new OMPs to be approved in Europe [27]. Under the ‘optimistic’ scenario (higher number of approved OMPs) the authors predicted that there would be more than 200 approved OMPs by 2020, against 152 predicted in this study. However the EURORDIS study is likely to have overestimated the success rate given that it predicted over 120 approved OMPs by the end of 2012, where in fact only 78 were approved by this time [4].

The price level and sales revenue for OMPs is important in determining the budget impact in future. The distribution of sales is skewed between OMPs, with a few drugs accounting for a large share of aggregate budget impact. The majority of OMPs have relatively low annual sales. The success of some outlier OMPs, such as imatinib, has contributed to an impression that OMPs consistently achieve high sales. In reality, between 2000 and 2012, only the top five selling OMPs in each market have reported average sales per annum in excess of SEK 30 million in Sweden and EUR 50 million in France [17,18].

It is possible that in future this situation might change. For example, it has been suggested that the greater participation of larger pharmaceutical manufacturers in the OMP market might lead to higher prices and increased volumes of sales, or changes in the political or payer environment could further affect uptake. While this is difficult to predict, sensitivity analysis suggested that an increase in average OMP sales by 50% would almost double the budget impact versus the base case. However such a large increase in average OMPs sales must be considered unlikely given the scrutiny that OMPs face during pricing negotiations at product launch [3].

The price change of OMPs at loss of IPP is also uncertain and important. It is too early in the history of European Regulation to know how OMP prices will change once IPP is lost. The impact of genericization on price is influenced by many factors, including the size of the patient population, the complexity of the manufacturing process, and the number of generic manufacturers who enter the market [28]. Imatinib, which accounted for 34% of total OMP sales between 2000 and 2012 in Sweden, and for 27% of total OMP sales in France [17,18], became subject to generic competition in late 2013, with four generics being approved in Europe [29]. While it is too early to know the effect this will have on price, the extent of interest from generic manufacturers is positive. The conservative assumption in the sensitivity analysis that loss of IPP will have no impact on price is unlikely [30]. Regardless, the results of the analysis were not particularly sensitive to assumptions concerning price change at loss of IPP.

Results from this analysis also match previous results by from the European-wide budget impact assessment of OMPs carried out by the same authors in 2011 [13]. Despite a different modeling approach that used rare diseases rather than OMPs as the primary forecasting unit, the previous analysis predicted a peak budget impact of 4.6% of total European pharmaceutical expenditure, compared with 4.1% in Sweden and 4.9% in France in the current analysis. These findings support the structural validity of the model and the assumptions used for this analysis.

The robustness of the forecasting model is further supported by the comprehensive nature of the data used. Average OMP sales were estimated using sales data from each country for all OMPs approved in Europe since the introduction of the OMP Regulation. Analyses on OMP designations and marketing authorization approvals used the cumulative history of such decisions since 2000, as well as further validating these data with similar comparisons using the longer term more extensive and robust US dataset.

Potential weaknesses in the model primarily relate to those associated with forecasting assumptions. The success rate for designated OMPs significantly affects the likely budget impact, but can only be predicted based on observed rates. These have changed over time, both in Europe and in the US, and this analysis assumes a constant and recent European trend rate. In reality, the US experience suggests that the future trend might be lower, meaning that assumptions for this analysis may be conservative. Furthermore, it is impossible to precisely estimate how the denominator in the model – the growth of the wider pharmaceutical market in both countries – will evolve in the forthcoming years. Demographic change is by itself likely to result in continued drug expenditure growth in future. The 2% growth rate used in the base-case is half the average growth rate observed in the last 12 years (Figure 7), reflecting the lower GDP growth in Europe. Sensitivity analyses confirmed that the model was not sensitive to this parameter.

The base-case results from this model were implicitly compared against the linear trend rate of the OMP budget impacts from France and Sweden from 2000–2012. The authors believe that the more sophisticated modeling exercise conducted in this analysis has greater validity because it incorporates additional available information relating to future OMP designations, marketing authorization approvals and price adjustments following loss of IPP. Critically, given that all OMPs are covered by a 10-year marketing exclusivity, a simple extrapolation from the first decade of OMP experience in these countries would neglect the impact of loss of IPP from marketed OMPs.

## Conclusions

The budget impact of OMPs in France and Sweden is expected to plateau between 4% and 5% of the total national pharmaceutical market expenditure in 2020, and is therefore likely to remain sustainable and a small proportion of total pharmaceutical expenditure, despite payer concerns about growing designation rates. Expected sustainable expenditure growth reflects low success rates for OMP marketing approval, and the impact of IPP expiries. This analysis represents the most robust assessment to date of the future budget impact of OMPs in France and Sweden.

## Competing interests

Adam Hutchings, Carina Schey and Richard Dutton have provided consulting services to pharmaceutical companies, including Celgene Corporation.

Felix Achana was reimbursed by GMAS LLP for statistical services provided in support of this research.

Karolina Antonov is employed by LIF, the Swedish Pharmaceutical Association.

## Authors’ contributions

AH and CS were involved in the conception of the study, the development of the analytical model, the interpretation of the results and the writing of the manuscript. RD was involved in the conception of the study and the development of the analytical model. FA undertook the statistical analysis of historical sales data. KA was involved in obtaining and validating data used in the model and in the interpretation of the results. All authors read and approved the final manuscript.

## References

[B1] EURORDIS About rare diseases http://www.eurordis.org/about-rare-diseases

[B2] Regulation (EC) No 141/2000 of the European parliament and of the council of 16 December 1999 on orphan medicinal products http://eur-lex.europa.eu/LexUriServ/LexUriServ.do?uri=OJ:L:2000:018:0001:0005:en:PDF

[B3] Sharma A Jacob A Tandon M Kumar D Orphan drug: development trends and strategies J Pharm Bioallied Sci 2010 2 290 299 10.4103/0975-7406.72128 21180460 PMC2996062

[B4] European Commission Community register of medicinal products 2013 http://ec.europa.eu/health/documents/community-register/html/orphreg.htm. Analyzed May 2013

[B5] Simoens S Pricing and reimbursement of orphan drugs: the need for more transparency Orphanet J Rare Dis 2011 6 42 10.1186/1750-1172-6-42 21682893 PMC3132155

[B6] Denis A Mergaert L Fostier C Cleemput I Simoens S A comparative study of European rare disease and orphan drug markets Health Policy 2010 97 173 179 10.1016/j.healthpol.2010.05.017 20800761

[B7] Roos JC Hyry HI Cox TM Orphan drug pricing may warrant a competition law investigation BMJ 2010 341 c6471 10.1136/bmj.c6471 21081598

[B8] Sheldon T Dutch doctors call for EU evaluation of cost effectiveness of high cost orphan drugs BMJ 2012 345 e5461 10.1136/bmj.e5461 22890124

[B9] Denis A Mergaert L Fostier C Cleemput I Simoens S Budget impact analysis of orphan drugs in Belgium: estimates from 2008 to 2013 J Med Econ 2010 13 2 295 301 10.3111/13696998.2010.491427 20482245

[B10] Orofino J Soto J Casado MA Oyaguez I Global spending on orphan drugs in France, Germany, the UK, Italy and Spain during 2007 Appl Health Econ Health Policy 2010 8 301 315 10.2165/11531880-000000000-00000 20804223

[B11] Picavet E Annemans L Cleemput I Cassiman D Simoens S Market uptake of orphan drugs–a European analysis J Clin Pharm Ther 2012 37 664 667 10.1111/j.1365-2710.2012.01364.x 22731105

[B12] Bignami F Eurordis survey on orphan drugs availability in Europe. 6th eurordis round table of companies workshop, Barcelona, 9 July 2007 2007 http://www.eurordis.org/IMG/pdf/2007ODsurvey-eurordis.pdf

[B13] Schey C Milanova T Hutchings A Estimating the budget impact of orphan medicines in Europe: 2010–2020 Orphanet J Rare Dis 2011 6 62 10.1186/1750-1172-6-62 21951518 PMC3191371

[B14] Le Cam Y Inventory of access and prices of orphan drugs across Europe http://img.eurordis.org/newsletter/pdf/mar-2011/ERTC_13122010_YLeCam_Final.pdf

[B15] Garau M Mestre-Ferrandiz J Access mechanisms for orphan drugs: a comparative study of selected European countries OHE Briefing 2009 52 1 24

[B16] Habl C Bachner F EMINET. Initial investigation to assess the feasibility of a coordinated system to access orphan medicines Updated Final Report 2011 Vienna: Commissioned by the European Commission, Directorate-General Enterprise and Industry

[B17] Intercontinental Medical Statistics (IMS) Health database 2013 MIDAS Data on file

[B18] Groupement pour l’Elaboration et la Réalisation de Statistiques (GERS) 2013 Data on file

[B19] Orphan drugs US food and drug administration 2013 http://www.accessdata.fda.gov/scripts/opdlisting/oopd/OOPD_Results_2.cfm] Data on file. Analyzed March 2013

[B20] US Food and Drug Administration Developing Products for rare Diseases and Conditions http://www.fda.gov/ForIndustry/DevelopingProductsforRareDiseasesConditions/default.htm

[B21] Lunn DJ Thomas A Best N Spiegelhalter D WinBUGS – a Bayesian modelling framework: concepts, structure, and extensibility Stat Comput 2000 10 325 337 10.1023/A:1008929526011

[B22] Patent and marketing exclusivity analysis 2012 Celgene Data on file

[B23] TLVFS 4 Tandvårds- och läkemedelsförmånsverkets föreskrifter och allmänna råd om prissättning av utbytbara läkemedel och utbyte av läkemedel m.m 2009 http://www.tlv.se/Upload/Lagar_och_foreskrifter/tlvfs-2011-4.pdf

[B24] Comite Economique Des Produits De Sante Rapport d’activitite 2012 2013 http://www.sante.gouv.fr/les-activites-du-ceps.html

[B25] Tandvårds- och läkemedelsförmånsverket (TLV) http://www.tlv.se/apotek/apotekets-marginaler/%5D

[B26] Orphanet. Orphan Drugs. The number of orphan medicine designations passes the 1000 milestone in Europ 2012 http://www.orpha.net/actor/EuropaNews/2012/120620.html

[B27] EURORDIS Orphan drugs: rising to the challenge to ensure a better future for 30 million patients in Europe 2009 http://www.eurordis.org/sites/default/files/publications/Statement_Future_of_Orphan_Drugs_14_October_09.pdf

[B28] Rovira J Espín J García L Olry de Labry A The impact of biosimilars’ entryin the EU market 2011 http://ec.europa.eu/enterprise/sectors/healthcare/files/docs/biosimilars_market_012011_en.pdf

[B29] European medicines agency http://www.ema.europa.eu/ema/index.jsp?curl=search.jsp&q=imatinib&btnG=Search&mid=WC0b01ac058001d124

[B30] European commission. Pharmaceutical sector inquiry. Preliminary report 2008 http://ec.europa.eu/competition/sectors/pharmaceuticals/inquiry/preliminary_report.pdf

